# Modified anchor traction method allows safe colorectal endoscopic submucosal dissection: the T-shaped traction method

**DOI:** 10.1055/a-2512-4297

**Published:** 2025-01-28

**Authors:** Keisaku Yamada, Masahiro Tajika, Tsutomu Tanaka, Nobuhito Ito, Akihiro Takagi, Yasumasa Niwa

**Affiliations:** 1 Department of Endoscopy, Aichi Cancer Center Hospital, Nagoya, Japan

Using a traction device can reduce the procedure time and complications associated with colorectal endoscopic submucosal dissection (ESD) 1 2 . We developed a novel traction technique using a multi-loop traction device (MLTD; Boston Scientific, Tokyo, Japan) that enables traction at three locations; we termed this the “anchor traction method” 3 . However, with this traction method, attachment to the opposite side of the intestinal tract may be difficult in places where the lumen is wide, such as in the cecum and ascending colon, because of the shortness of the traction device, and there may be a risk of perforation caused by elevation of the muscle layer due to excessive traction force (pulling too hard). Here, we report a useful traction method that overcomes these points by combining two MLTDs in colorectal ESD.

An 84-year-old woman presented with a 35-mm type 0-IIa lesion in the cecum ( Fig. 1 ), and underwent ESD ( Video 1 ). A full circumferential incision was made, the middle loop of the MLTD was attached to a reopenable clip (SureClip; MicroTech, Nanjing, China), and the two remaining loops of the MLTD were then attached to the lesion as in the previously reported anchor traction method. Next, a new MLTD with a clip was delivered and that clip was attached to the middle loop of the first MLTD. Good traction was then obtained by attaching the opposite loop of the second MLTD to the intestinal mucosa on the opposite side of the lumen ( Fig. 2 ). In this way, the submucosa became clearly visible, allowing ESD with a knife to be performed safely. Pathological analysis revealed that the lesion was an intramucosal carcinoma measuring 30 × 25 mm with negative margins ( Fig. 3 ).

**Fig. 1:**
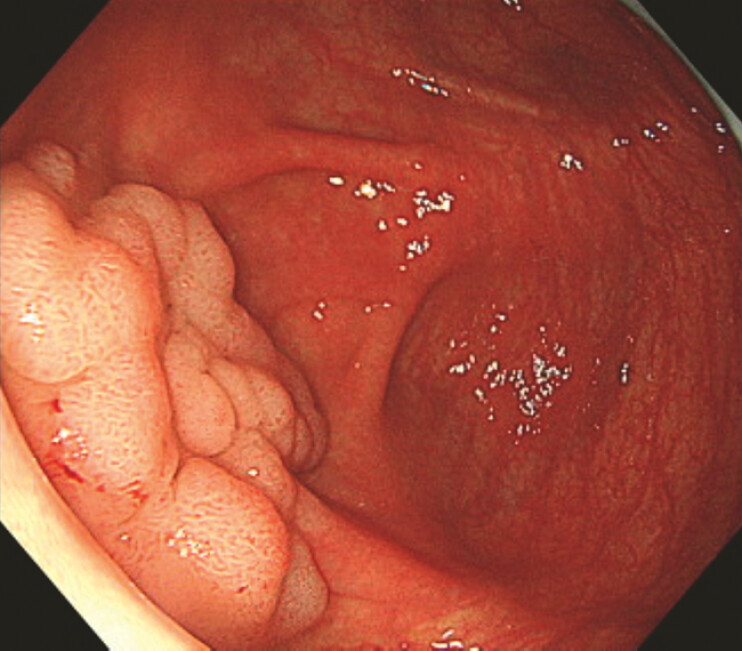
In an 84-year-old woman, a 35-mm type 0-IIa lesion at the cecum.

**Fig. 2:**
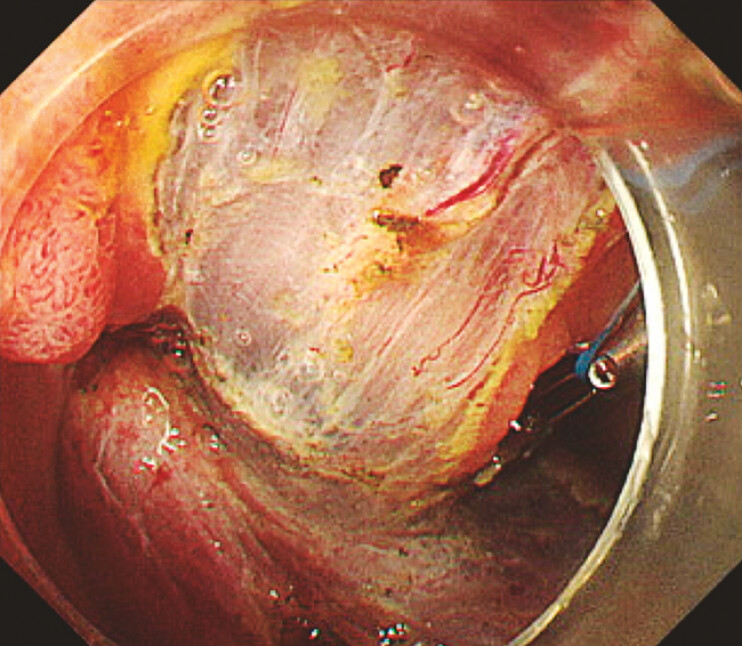
The T-shaped traction method improved the visibility of the submucosal layer.

**Fig. 3:**
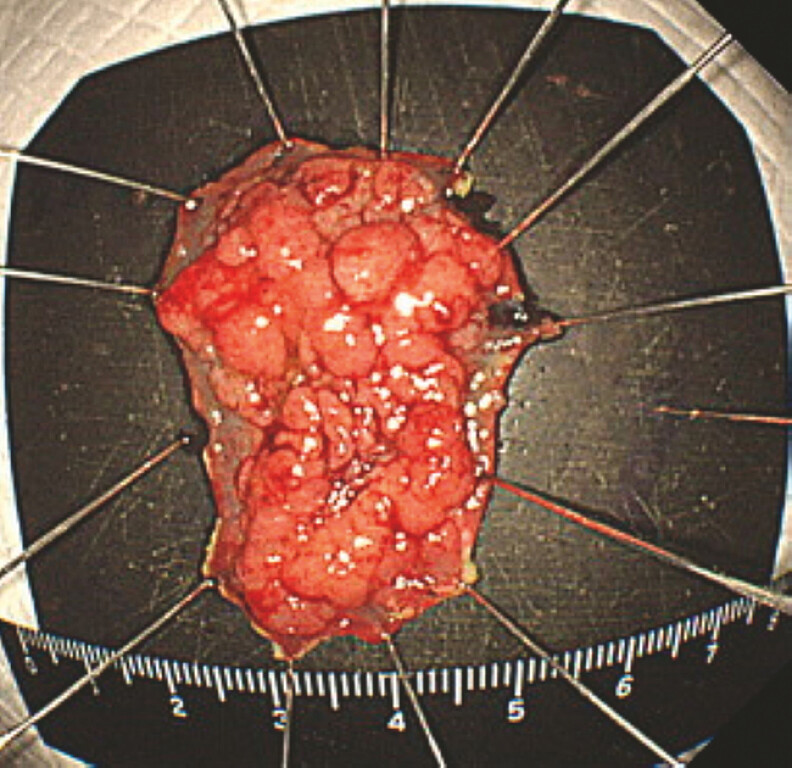
Histopathology showed the lesion to be adenocarcinoma in adenoma with negative margins.

T-shaped traction method allows safe colorectal endoscopic submucosal dissection.Video 1

This traction method combines two MLTDs to enable traction at multiple locations safely without excessive traction on the muscle layers even in a wide lumen; it is called the “T-shaped traction method.”

Endoscopy_UCTN_Code_TTT_1AQ_2AD_3AD

## Notes

Endoscopy E-Videos https://eref.thieme.de/e-videos
*E-Videos* is an open access online section of the journal *Endoscopy* , reporting on interesting cases and new techniques in gastroenterological endoscopy. All papers include a high-quality video and are published with a Creative Commons CC-BY license. Endoscopy E-Videos qualify for HINARI discounts and waivers and eligibility is automatically checked during the submission process. We grant 100% waivers to articles whose corresponding authors are based in Group A countries and 50% waivers to those who are based in Group B countries as classified by Research4Life (see: https://www.research4life.org/access/eligibility/ ). This section has its own submission website at https://mc.manuscriptcentral.com/e-videos .
